# Synaptic Interactions in Germinal Centers

**DOI:** 10.3389/fimmu.2018.01858

**Published:** 2018-08-13

**Authors:** Ilenia Papa, Carola G. Vinuesa

**Affiliations:** John Curtin School of Medical Research, Australian National University, Acton, ACT, Australia

**Keywords:** T follicular helper (T_FH_) cell, germinal center, germinal centre B cells, immunological synapse, dense core granules

## Abstract

The germinal center (GC) is a complex, highly dynamic microanatomical niche that allows the generation of high-affinity antibody-producing plasma cells and memory B cells. These cells constitute the basis of long-lived highly protective antibody responses. For affinity maturation to occur, B cells undergo multiple rounds of proliferation and mutation of the genes that encode the immunoglobulin V region followed by selection by specialized T cells called follicular helper T (T_FH_) cells. In order to achieve this result, the GC requires spatially and temporally coordinated interactions between the different cell types, including B and T lymphocytes and follicular dendritic cells. Cognate interactions between T_FH_ and GC B cells resemble cellular connections and synaptic communication within the nervous system, which allow signals to be transduced rapidly and effectively across the synaptic cleft. Such immunological synapses are particularly critical in the GC where the speed of T–B cell interactions is faster and their duration shorter than at other sites. In addition, the antigen-based specificity of cognate interactions in GCs is critical for affinity-based selection in which B cells compete for T cell help so that rapid modulation of the signaling threshold determines the outcome of the interaction. In the context of GCs, which contain large numbers of cells in a highly compacted structure, focused delivery of signals across the interacting cells becomes particularly important. Promiscuous or bystander delivery of positive selection signals could potentially lead to the appearance of long-lived self-reactive B cell clones. Cytokines, cytotoxic granules, and more recently neurotransmitters have been shown to be transferred from T_FH_ to B cells upon cognate interactions. This review describes the current knowledge on immunological synapses occurring during GC responses including the type of granules, their content, and function in T_FH_-mediated help to B cells.

## Immune Synapse: Principles, Organization, and Structure

The term synapse was first used to describe the typical neural connections in the nervous system, which allow transmission of an electrical or chemical signal from one neuron to a responding cell in close physical contact. Immunologists then co-opted the term and referred to “immunological synapse” to describe the interactions between an antigen-presenting cell (APC) and an antigen-receptor expressing immune cell that involve close contact and the release of molecules such as cytokines across the synaptic space (1, 2).

When naïve T cells recognize peptide-MHC on APCs *via* their TCRs, the TCRs become organized into structures of ~500 nm known as microclusters (MCs). These MCs are more efficient in the recruitment of kinases and adapters that can initiate an activation signaling cascade (3). During formation of the immunological synapse, the TCR-MCs localize at the center of the interface between the T cells and the APC giving rise to the central supramolecular activation cluster (cSMAC) (4–7). This cSMAC is also called the bull’s eye-type immunological synapse, due to its characteristic appearance, as first described by Kupfer (8). The immunological synapse between a T cell and an APC requires close juxtaposition of the membranes from the two different cell types. This is facilitated by a kinetic segregation of molecules that excludes negative regulatory phosphatases such as CD45 that relocates to the most external region or distal SMAC, and allows concentration of the key TCR signaling molecules at the center. This segregation process has been suggested to be an integral part of immune synapse function (9).

Besides TCR signaling, integrins play a key role in T cell activation facilitating the formation of conjugates between T cells and APCs. Lymphocyte function-associated antigen-1 (LFA-1) is one of the most important integrins during the process of T cell activation. LFA-1 and its high-affinity ligand intercellular adhesion molecule 1 (ICAM-1), localize outside of the cSMAC, at the peripheral SMAC (pSMAC). The inside-out signal from TCR or chemokine stimulation elicits conformational changes in LFA-1 that increase affinity for its ligands and therefore adhesion between the interacting cells (10). Binding of LFA-1 by ICAM-1, then leads to what is known as “outside-in” signaling, which contributes to many aspects of T cell activation.

Most membrane-proximal signaling molecules crucial for T cell activation such as ZAP70, LAT, SLP76, PLC-γ, etc., are recruited to TCR-MCs. Regulation of these large protein-complexes determines the outcome of T cell activation, not just in terms of TCR signaling strength but also with regards to the nature of the resulting effector cells (7, 11). It is still unclear how different activation, differentiation, and survival outcomes can derive from changes in the signal strength downstream of these signaling complexes.

Together with T-cell antigen receptors and integrins, two additional groups of receptors are located at the synapse: adhesion and costimulatory receptors. Adhesion is mediated by heterophilic interactions between the signaling lymphocyte activation molecules (SLAM) family members CD2 (expressed on T cells) and CD58 (expressed on APCs). These CD2–CD58 interactions can contribute to TCR signaling processes even when direct TCR stimulation is absent (12).

It has been known for over two decades that costimulatory receptors are poor in eliciting activation signals or inducing cell adhesion on their own, but when combined with signals from other receptors, most prominently the TCR, they can potently enhance T cell activation, adhesion, and differentiation (13–15). The typical T cell costimulator is CD28, a member of the Ig superfamily characterized by a homodimeric structure and a cytoplasmic domain. The cytoplasmic domain of CD28 recruits and activates Lck, which can then phosphorylate and activates protein kinase C (PKC)-θ. In T cells PKC-θ, a critical PKC isoform, contributes to the activation of NF-κB transcription factors and promotes IL-2 production (16). Ligation of B7-1 (CD80) and B7-2 (CD86) on APCs and interaction within an immunological synapse regulate CD28 activity (17). Upregulation of CD80 and CD86 on DCs is a downstream effect of toll-like receptors signals and inflammatory cytokines (18, 19). In addition, expression of the inducible T cell costimulator, ICOS on activated T cells helps recruitment of the p50α PI3K regulatory subunit to the immunological synapse, resulting in stronger activation of PI3K (20).

## B Cell–Follicular Dendritic Cell (FDC) Synapses

Synaptic interactions between B cells and FDCs are key for B cells to efficiently extract antigen held in the form of immune complexes on FDCs, and to promote B cell survival until T_FH_ selection and survival signals are delivered. Only those germinal center (GC) B cells able of binding and taking up antigen from FDCs to then present processed peptide to T_FH_ cells can survive and differentiate into memory B cells or plasma cells. Immune complexes in association with activated complement components are bound by immunoglobulin receptors, CD35 and CD21 expressed on FDCs. Interaction between the BCR and antigen held in these immune complexes induces BCR signaling, BCR–antigen MC formation followed by the formation of a mature immune synapse and antigen internalization for subsequent processing and presentation to T cells (21–23).

*In vitro* studies suggested that GC B cells form unique synaptic structures compared to other B cell subsets (24). GC B cells form synapses containing less antigen than naive B cells, and the antigen is confined preferentially to the pSMAC rather than being localized at the cSMAC. In addition, some GC B cells form small synapses using their characteristic and previously described lamellipodia-like protrusions (24–26).

The early observation that B cells can acquire antigen that is bound to a surface suggested that mechanical forces are required for this process (21). Subsequently, it has been demonstrated that B cells are able to pull and internalize antigens (24), this process is dependent on the nature of the antigen nature and on the physical characteristics of antigen presentation (27). Stiff substrates, such as the FDC membrane, allow higher affinity discrimination, whereas antigen extraction from flexible substrates is more efficient. These findings, together with the observation that GC B cells require stronger forces in order to pull and take up antigen from the synaptic interface compared to naive B cells (24), support the importance of synaptic interactions between FDCs and GC B cells in affinity maturation and antibody production.

**Figure 1:**
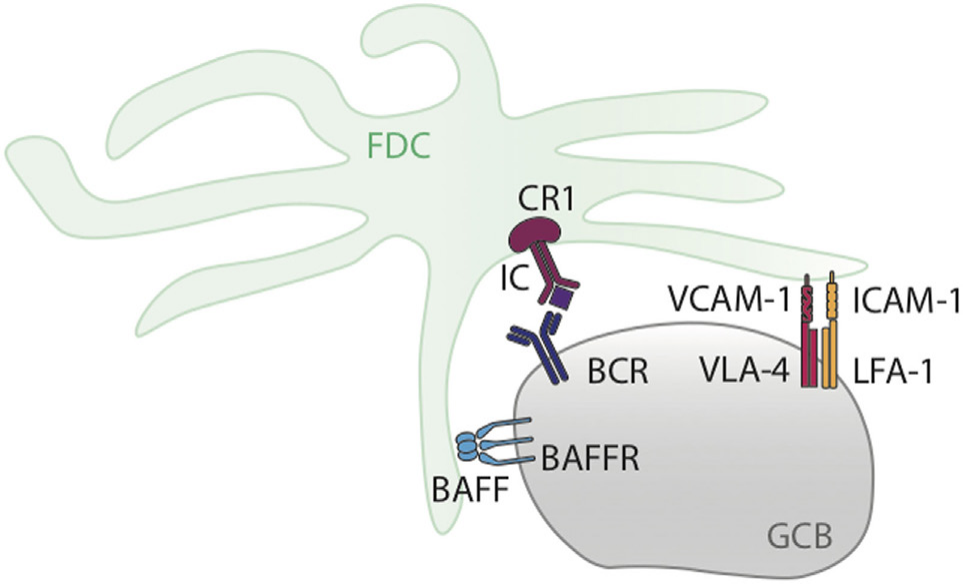
Graphical representation of GC B cell–follicular dendritic cell (FDC) interaction in the germinal center (GC). The diagram shows key molecules involved in the interactions between GC B cells and FDCs happening during GC responses and discussed in Section “B Cell–Follicular Dendritic Cell (FDC) Synapses.”

The synaptic interactions between GC B cells and FDCs involve several molecules (Figure 1). The first group comprises adhesion proteins such as B cell expressed LFA-1 that interacts with ICAM-1 on FDCs, and very-late activation antigen 4 (VLA-4) that interacts with VCAM-1 (28). These molecules do not deliver directly anti-apoptotic signals, but promote the anti-apoptotic functions of FDCs by augmenting cell–cell contact. Of note, GC B cells undergo apoptosis when separated from FDCs.

Expression of VCAM-1 and ICAM-1 on FDCs relies on NF-kB signaling (29) downstream of FCγRIIB, as Fcgr2b^−/−^ mice fail to upregulate *Icam1* or *Vcam1* mRNA and protein on FDCs after immunocomplex formation (30). In mice lacking NF-κB signaling in FDCs, GCs were smaller and contained more apoptotic cells. Also primary responses to sheep red blood cells were partially reduced and secondary immunizations did not induce strong responses (29), although this could be due to other processes rather then a direct effect of ICAM-1/VCAM-1 regulation.

*In vitro* studies have suggested that the integrins ICAM-1 and VCAM-1 may play a role in GC B cell survival. Indeed, Lindhout et al. showed that direct interaction between FDCs and human GC B cells facilitated GC B cell survival in culture (31). Subsequent studies revealed that equivalent survival could be achieved when replacing FDCs by coating VCAM-1 and ICAM-1 to the culture plates (28). Rapid death of GC B cells has been shown after diphtheria toxin-mediated ablation of FDCs *in vivo*, supporting a role of FDCs in promoting GC B cell viability (32). It has also been shown *in vivo* that mutation in DOCK8, member of a family of proteins critical for the activation of the Rho family of small GTPases, lead to disrupted concentration of ICAM-1 on B cells forming immune synapses, affecting their survival and affinity maturation (33). Nevertheless, a separate study showed minimal impact of ICAM1 and VCAM1 loss from FDCs on the magnitude of the GC response *in vivo* (34), which questions the importance of FDC-expressed VCAM1 and ICAM1 in GC B cell survival. It is possible that these interactions play a more significant role under some immunization conditions and may differentially impact the plasma cell or memory B cell response.

The second group of molecules influencing FDC–GC B cell interactions comprises pro-survival molecules and growth factors. The B cell survival factor BAFF—also known as BLys—is produced at high amounts by activated FDCs. Despite its well-described role in the survival of peripheral B cells, it is still unclear whether BAFF plays a role in GC B cell survival. *In vivo* studies describing GC formation with relatively preserved affinity maturation in BAFF-deficient mice suggest it is dispensable (35–37). BAFF/BAFF-R signaling does, however, appear to have a function in GC maintenance, as in the absence of this factor, GCs disappear a few days after their formation. Lack of BAFF also leads to failure of FDC maturation and as a consequence, stimulation of B cells through immune complexes cannot occur (35, 37). It is plausible this FDC phenotype may be an indirect effect of B cell lymphopenia in the absence of BAFF. In BAFF-R defective A/WySnJ mice, the FDC reticulum was normal and GCs could form, but proliferation of GC B cells was impaired (35). BAFF also signals through the receptors TACI and BCMA and mice deficient in these receptors displayed normal GC formation (38, 39) suggesting BAFF-R signaling is responsible for the observed effects of BAFF on GC B cells. However, some B cells in A/WySnI mice (lacking BAFF-R) are able to mature, enter the GC reaction and support FDC maturation, whereas B cells from BAFF-deficient mice cannot (40), suggesting that in the absence of BAFF-R, the other receptors for BAFF and/or APRIL (BCMA or TACI) may take over the B cell maturation function of BAFF-R, although they do not play an essential role in GC maintenance.

Besides the production of survival factors, FDCs are known for their expression of transmembrane molecules such as the transcobalamin receptor 8D6, and cytokines including IL-6 and IL-15 (41, 42). These surface-expressed or secreted products can also participate in the growth of GC B-cells. 8D6, also known as or CD320 can promote GC B cell growth. 8D6 also appears to support the proliferation of plasma cell precursors generated by IL-10, enhancing antibody secretion (42, 43). Human FDCs also produce IL-15, and *in vitro* its membrane-bound form has been shown to signal through IL-2/IL-15Rβ (44, 45) to enhance proliferation of GC B cells. This proliferative effect has not been observed for IL-6, although this cytokine is required for proper GC formation (46). It is possible, however, that the effect of IL-6 on GC B cells is indirect, through its well-demonstrated role in promoting T_FH_ cell induction and maintenance (47, 48).

Besides their effects on GC B cells, FDCs also regulate T_FH_ cells. FDC-derived IL-6 has also been suggested to be important for T_FH_ cell maintenance (47). Interactions between T_FH_-expressed TIGIT (49, 50) and its receptor on FDCs—the high-affinity poliovirus receptor (CD155) (49, 50) may also contribute to T_FH_ regulation by FDCs during thymus-dependent (TD) B cell responses. cT_FH_ TIGIT^+^ cells have been shown to exhibit strong B cell helper functions, inducing plasma cell differentiation and immunoglobulin production (51). Engagement of TIGIT by CD155 promotes IL-10 while it restrains IL-12 production by DCs, leading to reduced T cell activation *in vitro* (52).

## T_FH_–GC B Synapses

**Figure 2:**
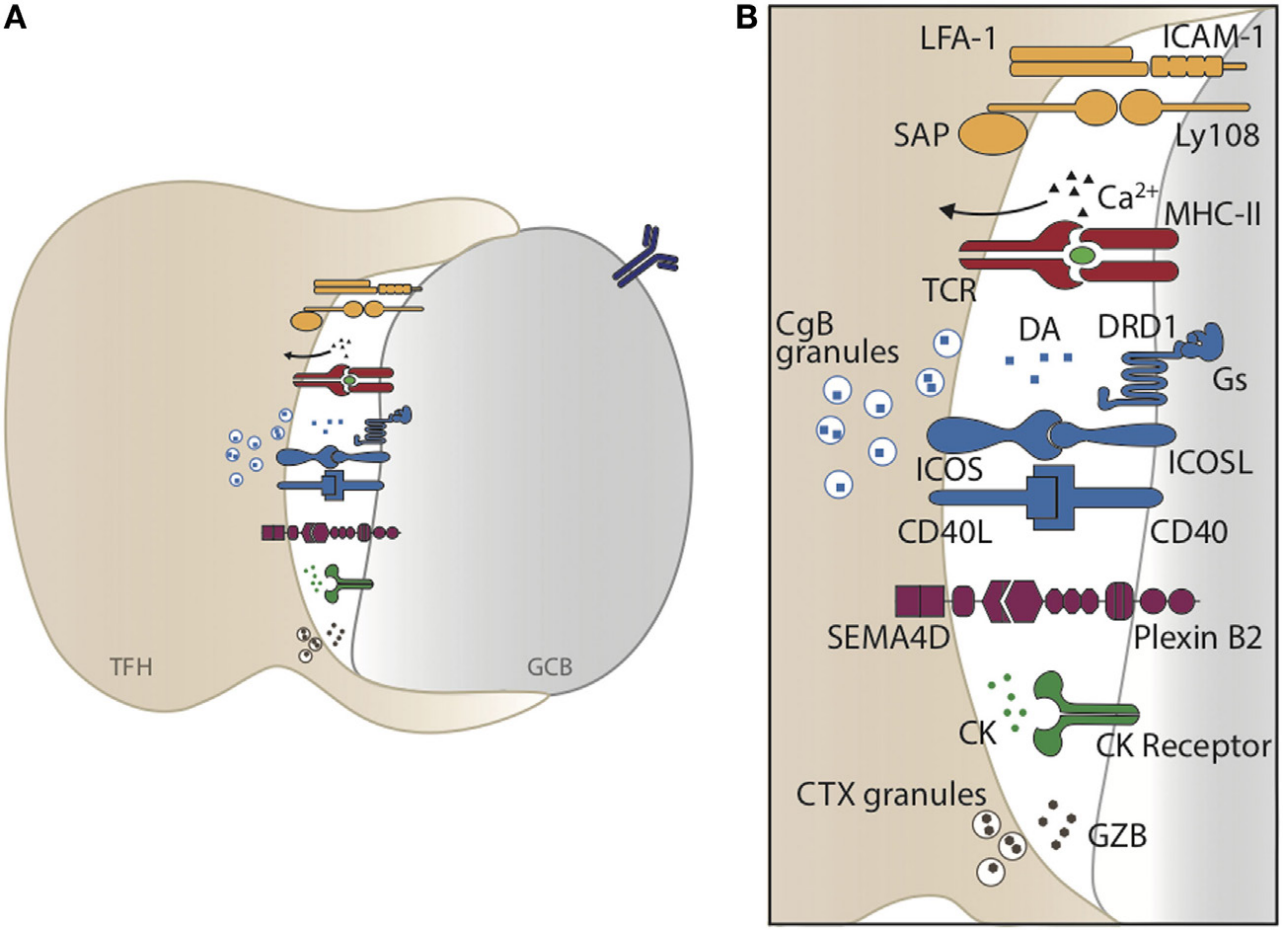
T_FH_–germinal center (GC) B cell synaptic interaction. **(A)** Graphical representation of T_FH_ and GC B cell interaction. Formation of optimal T_FH_–GC B cell synapses depends on specific interactions between receptor:ligand pairs and integrins, leading to a specific and focused transmission of cytokines, neurotransmitters, and cytotoxic granules. **(B)** Enlarged representation of the synaptic cleft forming upon T_FH_–GC B cell interaction.

Synaptic interactions between T_FH_ and GC B cells are essential for GC formation and GC B cell selection as they facilitate the delivery of T cell-derived helper molecules. Formation of optimal T_FH_–GC B cell synapses depends on specific interactions between the receptor:ligand pairs described below and summarized in Figure 2.

## ICAM-1:LFA-1

Intercellular adhesion molecule 1 also known as cluster of differentiation 54 is a ligand for the integrin LFA-1 and this pair forms the peripheral ring (pSMAC) of immune synapses. As mentioned above, during T cell-APC synapses, TCR activation upon binding peptide-MHC II molecules leads to a conformational change in LFA-1 and clustering of LFA-molecules. These structural and positional changes are critical to increase the affinity for ICAM molecules *via* the formation of multivalent associations (53). LFA-1 binding to ICAMs was also shown to be critical for adequate T cell activation and differentiation into effector cells (54, 55).

T_FH_ cells express high levels of LFA-1 (56) and Ag-specific B cells express higher levels of ICAM-1 compared to non Ag-specific cells (57). In addition to cognate pMHCII:TCR interactions, adhesive mechanisms and costimulatory receptors are critical for facilitating T–B conjugate formation and optimal TCR activation (54, 55, 58). Recently Zaretsky and colleagues showed that expression of two LFA-1 ligands on B cells—ICAM-1 and ICAM-2—was required to form the stable and lasting antigen-driven T_FH_–GC B cell interactions that promote B cell selection. Thus, the B cell antibody response to protein antigens depends on B cell ICAMs for optimal selection by T_FH_ cells. High levels of antigen can sustain short T–B interactions, however, optimal long-lasting contacts are strictly ICAM-dependent (57).

## Cell Surface-Polarized Stimulatory Molecules

### CD40:CD40L

T_FH_–GC B cell contacts require interactions between several key molecules. Among those, CD40L:CD40 interactions play crucial roles within GCs. Consequences of defective CD40–CD40L signaling highlight their important role in initiation and maintenance of GC responses. Blocking CD40 signals in established GCs leads to complete and rapid GC dissolution in mice (59). Indeed CD40 is critical for GC maintenance and enables light zone (LZ) cells to recycle back to the dark zone and sustain the GC response. In humans, CD40 deficiency causes hyper-IgM syndrome characterized by lack of switched memory B cells and switched serum immunoglobulins. CD40L was also shown to provide survival signals to human GC B cells (60). In mice, CD40 signals also induce ICOSL expression on GC B cells (61).

CD40L is found preformed in GC T cells (62) and is rapidly and transiently relocated on the T cell surface upon TCR ligation. There is recent evidence in mice that ICOS signals can contribute to CD40L upregulation. Indeed, as described above ICOSL signals delivered by GC B cells support the transient but extensive “entanglement” with T_FH_ cells, which in turn stimulates the increased expression of CD40L on the surface of T_FH_ cells (61). CD40L-mediated upregulation of ICOSL expression by GC B cells, followed by ICOSL induction of further CD40L expression on T_FH_ cells has been described as a feed-forward loop that enables high-affinity B cells to repeatedly acquire more T cell help than their lower affinity competitors (61).

Outside GCs it has been shown that both Th1 and Th2 cells transfer CD40L to B cells in an antigen-specific manner (63). Although cultured Th1 and Th2 cells have distinctive immunological synapse structures (64) with Th1 displaying a classical immune synapse and Th2 a multifocal synapse structure, both cell types are equally efficient in antigen-specific T–B interaction (63). Future analysis will be required to confirm the immunological synapse organization of primary Th1 and Th2 cells. In human primary T_FH_ cells, CD40L accumulated in the cSMAC on supported lipid bilayers containing CD40 and ICOSL (65), suggesting that T_FH_–GC B cell synapses display the structure of a classical immunological synapse.

### SLAM Family Members and SLAM-Associated Protein (SAP)

SLAM-associated protein expression in T cells has been shown to be important for adhesive cognate T–B cell interactions. In the absence of SAP, T–B interactions were less stable and were not long-lasting, impairing T_FH_ differentiation. This stabilization was shown to be a consequence of T-cell intrinsic signaling of SAP, which serves as an adaptor for the SLAM family of immune receptors at the cell–cell interface. B cells express high levels of several SLAM family members including SLAM, CD84, Ly9, and Ly108 molecules. These same molecules are also found in substantial amounts on the surface of activated T cells and in T_FH_ cells (66–70). Most SLAM family members bind in a homophilic fashion to the same molecule expressed on the interacting cell. SLAM molecules recruit SAP to their cytoplasmic tail leading to the downstream signaling cascade required to induce stable adhesive interactions (67, 71). *In vitro* experiments have proposed that CD84 and Ly108 act together during T–B cell synaptic interactions to promote the T_FH_ cell phenotype (66). SAP act as a break to Ly108-mediated recruitment of the inhibitory phosphatase SHP-1 to the T cell synapse. SHP-1 dephosphorylates immunotyrosine switch motifs normally bound by SAP, to limit T–B cell adhesion and prevent formation of sustained T–B cell synaptic interactions. The need of stable T–B cell interactions for an efficient GC response and production of disease-inducing autoantibodies has been also described in the context of autoimmunity (72). It is important to note that the role of SAP/SLAM in stabilizing cognate interactions between T cells and APCs appears to be essential in achieving sustained cognate T–B interactions, but not for T:DC interactions, which are more dependent on integrins (66).

T cell costimulatory ligands expressed by B cells also play key roles in T–B interactions and the resulting proliferation and differentiation of both T and B cells. CD80 and CD86 are markers of B cell activation and function providing important costimulatory signals *via* CD28 that promote T cell proliferation and cytokine production. CD86 is expressed by LZ B cells and promotes the APC capability of B cells (73). Reducing the availability of CD86 molecule by the injection of a blocking antibody (GL-1) during primary responses to NP-CGG resulted in the reduction of serum Ab titers, either IgM or IgG (59) whereas IL-21-mediated sustained elevation of CD86 augmented the magnitude of CD4 T cell responses both *in vitro* and *in vivo* (74). CTLA-4, which is found at high amounts on regulatory cells and some follicular T cells, can also bind to and transendocytose CD86 (75) but its function in GC reactions is still not clear.

### Plexin B2 (PlxnB2), Ephrin B1, and BASP1

Plexins constitute a family of transmembrane receptors for semaphorins and regulate multiple processes including synapse formation and axon guidance in the nervous system (76). PlxnB2 is expressed in the central nervous system upon binding semaphorins promotes axon guidance and migration (77). In the immune system, GC B cells formed in the context of TD but not thymus-independent responses express high amounts of PlxnB2 (25, 26). Recent studies have revealed that PlxnB2 expressed by GC B cells is sensed by Semaphorin 4C (Sema4C) expressed on T_FH_ cells. This PlxnB2–Sema4C interaction promotes T–B adhesion in an antigen-independent manner and guides T_FH_ cell recruitment to the GC (78). For this, PlxnB2 expressed on GC B cells promotes T_FH_ migration from the T zone to the border of the GC. Once positioned at the GC edge, T_FH_ cells have easier access into the GC. The evidence that this positioning is the critical first step comes from the observation that in mice lacking B cell expressed PlxnB2, T_FH_ cells accumulate at the edge of the GC. In these mice, GC T–B interactions are diminished resulting in poor GC-derived antibody responses, including the production of high-affinity antibodies and long-lived plasma cells (78).

In the GC, members of the Ephrin receptors family and their ligands also regulate cell migration and cell-to-cell interaction. Ephrin type-B receptor 4 (EPHB4) and EPHB6 are expressed on T_FH_ cells (79) and one of their ligands, Ephrin-B1 (EFNB1) has been found on GC B cells (79, 80). Ephrin ligands and their receptors are membrane-bound proteins that require direct cell–cell interaction to bind and activate downstream signaling pathways. EFNB1 suppresses GC B–T_FH_ cell adhesion, and mice lacking its expression showed reduction in plasma cell production and accumulation of IL-21_deficient T follicular helper cells within the GC (79). Intriguingly, EPNB1 has been reported to be expressed in a subset of GC B cells that share phenotypic features with memory B cells and are preferentially located in the LZ and outer areas of GCs. It is therefore possible that Ephrin interactions also regulate T_FH_–B cell interactions leading to memory B cell formation from GC B cells (80).

BASP1 is a myristoylated protein highly expressed in the brain and localized at the inner surface of the plasma membrane in presynaptic neurons. In planar lipid bilayers BASP1 can exert ion channel activity (81). In neurons, BASP1 mediates neurite outgrowth and axonal repair (82). BASP1 expression is absent on resting murine splenic B cells, but can be induced by B-cell activation with anti-IgM and anti-CD40 and is again selectively and strongly upregulated in TD GC B cells. BASP1 is therefore likely to control synaptic processes in GC B cells.

## Transmitted Molecules

### Delivery of Cytokines Across Immune Synapses

One of the major roles of both neurological and immunological synapses is the focused secretion of soluble components into the synaptic cleft where the secreted factors can achieve the desired concentration and selectively act on the precise post-synaptic neuron or antigen-specific lymphoid cell (83–85).

There is also increasing evidence of transfer of cellular contents from T cells to APCs in the form of microvesicles. This is a highly dynamic process by which microvesicles formed within the T cell are then released into the synaptic interface and taken up by APCs. Within APCs, the contents of microvesicles, which include proteins, RNAs, and microRNAs, can influence gene expression and activation of early signaling pathways (86, 87). Transfer in the opposite direction also occurs at the synaptic cleft: indeed, the process of trogocytosis, by which T cells can extract MHC:peptide complexes from APCs during endocytosis of engaged TCRs, has been shown to be important for sustained signaling at endosomes (88, 89). Together, these findings suggest that immune synapses are key facilitators of transcellular communication.

T_FH_-derived cytokines can be secreted and signal to B cells in a contact-independent manner. However, it seems that cell–cell contact can potently enhance the response. Indeed, secretion of cytokines across an immunological synapse can be highly effective due to the much higher concentrations that can be achieved within the synaptic space (90) are much more specific, because it is directed toward a B cell presenting cognate antigen. Variation in the amount of antigen presented can be detected in a highly sensitive manner, and the synapse can quickly change orientation to contact the cell presenting peptide:MHC complexes at the highest density. Together, these properties by which helper molecules get transmitted across an immune synapse are thought to enable T_FH_ cells to select those B cells expressing BCRs with the highest affinity for the immunizing antigen.

The observation made by Reinhardt and colleagues offers an example of this effective cytokine delivery. The isolation of T–B cell conjugates from the draining lymph node of immunized mice demonstrated that IgG1-producing B cells made contact with IL-4-producing T cells whereas IgG2a-producing B cells made contact with IFN-γ-producing T cells (91). IL-4-producing T cells were found conjugated to GC B cells expressing high levels of AID, with evidence of somatic hypermutation (91) demonstrating that T_FH_-derived cytokines directly stimulate the production of different antibody isotypes in responding B cells sharing the same microenvironment. However, additional studies would be required to confirm these observations, perhaps using the LIPSTIC method, which allows direct measurement of dynamic cell–cell interactions both *in vitro* and *in vivo* (92).

### Neurotransmitters in T–B Synapses

Neurotransmitters (NTs) are proteins used by the nervous system to communicate between neurons or other cells. This response typically occurs in response to changes in action potential when the neuron is activated. Substances acting as transmitters are stored in vesicles at synapses and are released by a process of exocytosis. Exocytosis in neurons occurs when depolarization of the neuron cell wall causes flux of calcium, binding of vesicles, and eventual externalization of vesicular content. Substances considered to be neurotransmitters are released into the synaptic cleft by exocytosis and/or directly from the cytoplasm. A neurotransmitter can be defined as a substance that is released by a neuron and that affects a specific target in a specific manner. A target can be either another neuron or an effector organ, such as muscle or gland. The concept of a transmitter is not precise, as neurotransmitters are protean, structurally resembling other released agents in many regards. NTs act on targets that are close to the site of transmitter release, in distinction to hormones that are released in the bloodstream to act on distant targets (93). The interaction of neurotransmitters with receptors is typically transient, lasting from milliseconds to minutes. Despite the short timeframe of interaction, neurotransmitter action can result in long-term changes within target cells lasting hours or days.

Dopamine (DA) is a catecholamine mainly synthetized in the central nervous system where it acts as a neurotransmitter. The rate-limiting step in catecholamine synthesis is the conversion of tyrosine into l-DOPA by the enzyme tyrosine hydroxylase. Dopamine is then synthetized from l-DOPA by the enzyme DOPA decarboxylase. In the presence of other enzymes, dopamine can be further converted into noradrenaline and adrenaline.

In neurons, dopamine is packaged into vesicles after synthesis and can be released into the synaptic cleft upon the occurrence of a presynaptic action potential. Neuronal cells can also secrete dopamine into peripheral tissues. Furthermore, dopamine can also be synthetized within specific parenchymal tissue and endothelial cells (94, 95). Nevertheless, despite evidence of endothelial and other sources of peripheral dopamine production, the main contributor to plasma dopamine levels is production by sympathetic nerves.

An emerging role for dopamine in the immune system has recently been recognized. Dopamine can be produced by immune cells such as T cells and dendritic cells, and its release has various autocrine and paracrine effects (96–107).

Only recently, a role for dopamine in the GC reaction has been described (65). High dopamine amounts are found in T_FH_ cells compared to other T cell subsets analyzed. Dopamine is typically stored in dense core granules. Chromogranin B (CgB) is a marker of dense core secretory granules in the neuroendocrine system and is involved in the packaging of catecholamines, such as dopamine and noradrenaline. CgB^+^ granules are found in a small percentage of human T_FH_ cells. T_FH_ cells can synthetize dopamine upon cAMP induction and release it during T–B cell synapse formation. Once released, dopamine can bind to dopamine receptors expressed by human GC B cells and induce ICOSL translocation to the cell surface within minutes of stimulation. This effect appears to be mediated by dopamine receptor 1 (DRD1). Ligation of ICOS on human T_FH_ cells leads to fast translocation of CD40L to the center of the synapse. Furthermore, ICOS ligation also augmented the area of the T_FH_–GC B cell synapse (65). This work adds to the growing evidence of the importance of ICOSL-mediated regulation of T cell help to GC B cells, required for productive GC reactions.

### Cytotoxic Granules and Granzymes

Cytolytic granules are specialized secretory lysosomes containing a set of proteins, such as perforin and granzymes, involved in cell-mediated apoptosis (108, 109). Cytolytic granules can also be delivered through the immunological synapse. Specifically, this occurs through a secretory zone localized in the center of the synapse (110). Although cytotoxicity is a typical property of CD8^+^ T cells and natural killer cells, MHC class-II-restricted cytotoxicity mediated by CD4^+^ T cells has also been described in both humans and mice (111–116). CD4^+^ CTL have been identified mostly during viral infections, suggesting that one of the main roles of CD4^+^ CTLs is antiviral immunity. CD4^+^ CTLs have also been identified during antitumor responses (117, 118) and chronic inflammatory responses (119–121).

Recent findings in mice described that the infecting or immunizing virus influences CD4^+^ CTL differentiation and that this differentiation program, once initiated, directly antagonizes T_FH_ differentiation. CD4 CTLs express high levels of Blimp1 and low levels of Bcl6, which is required for T_FH_ cell differentiation. Unlike the dependency of T_FH_ cells on BCL6 and TCF1, these transcription factors prevent CD4^+^ CTL induction, suggesting a dichotomous differentiation pathway between CD4^+^ T_FH_ and CTLs (122).

In human GCs a subset of T_FH_ cells expressing the surface marker CD57 (HNK-1/Leu-7) show cytotoxic activity (123). CD57 is generally upregulated in cells with cytotoxic activity (124). Further characterization of the nature and function of these granules will be required.

## Consequences of T–B Synaptic Interactions

In the nervous system, calcium fluxes are essential facilitators of synaptic neurotransmission. Action potentials open calcium channels in the presynaptic membrane causing the uptake of calcium ions (Ca^2+^). This calcium flux triggers the release of neurotransmitters from synaptic vesicles into the synaptic cleft. Calcium fluxes have been well studied in the context of T cell-APC synapse formation. It is only recently that calcium mobilization has been observed in the context of T_FH_–GC B cell engagement.

Selection of high-affinity antibody-producing B cells is mediated by large but transient interactions between T_FH_ and GC B cells. It has been shown that in the presence of antigen, T cells reduce their speed and increase the duration and area of contact with high-affinity GC B cells. These interactions lead to an increase in T_FH_ intracellular calcium, which in turn increases the amount of helper cytokines IL-4 and IL-21 (125). Subsequent studies showed that mouse GC T cells help B cells in GCs *via* formation of entangled contacts, require extensive T and B cell surface interactions and rapid CD40L translocation to the surface of T_FH_ cells. This translocation of preformed CD40L requires ICOS costimulation and calcium signaling (61). When ICOSL knockout B cells were engaged in interactions with T_FH_ cells, smaller calcium fluxes in the T cells were detected.

Ca^2+^ mobilization is also important in B cells after BCR engagement leading to different outcomes depending on the presence of additional signals. BCR engagement alone by antigen triggers a Ca^2+^ flux that causes downregulation of constitutive ICOSL surface expression on activated B cells (126, 127) and *in vitro* stimulated GC B cells (65). This downregulation is potentiated when BCRs are engaged together with IL4R activation, which acts through STAT6 to cause complete loss of ICOSL surface expression. By contrast, costimulation of B cells through CD40 signals (but not LPS or cytokines) after downregulation by antigen and/or IL-4 could restore expression of ICOSL. Restoration of ICOSL expression did not occur in B cells treated with anti-IgM. This was thought to be a consequence of high BCR crosslinking, since HEL stimulation, which does not crosslink BCRs, did not prevent ICOSL re-expression. Together these findings highlight that CD40–CD40L signaling and the nature of the antigen, control whether antigen-activated B cells are able to re-express ICOSL, which in turns leads to costimulation of the cognate T cell (126).

## Concluding Remarks

The last decades of research in T–B cell interactions have revealed the importance of selective and focused delivery of important signals through the immunological synapse. Synaptic transmission of cytokines and neurotransmitters enables rapid regulation and translocation of molecules to the synaptic interface, required for effective T_FH_-mediated B cell selection. Several molecules are known to be involved in the initiation of T–B immune synaptic transmission in GCs. Less is known about the signals required for the termination of T–B cell interactions, or about signals that distinguish T_FH_–B vs T_FR_–B cell interactions. We expect the coming years will uncover a much larger array of neurotransmitter-like molecules involved in T–B interactions in GCs.

## Author Contributions

IP and CV conceived, designed, and wrote the manuscript. CV revised the manuscript.

## Conflict of Interest Statement

The authors declare that the research was conducted in the absence of any commercial or financial relationships that could be construed as a potential conflict of interest.
